# 742. Strain Replacement of Vancomycin-Resistant *Enterococcus faecium* over 6 years at a Large Tertiary Care Center

**DOI:** 10.1093/ofid/ofad500.803

**Published:** 2023-11-27

**Authors:** Emma Mills, Marrisa P Griffifth, Vatsala Rangachar, Kady Waggle, Lora Pless, Graham M Snyder, Alexander Sundermann, Lee Harrison, Daria Van Tyne

**Affiliations:** University of Pittsburgh, Pittsburgh, Pennsylvania; University of Pittsburgh, Pittsburgh, Pennsylvania; University of Pittsburgh, Pittsburgh, Pennsylvania; University of Pittsburgh, Pittsburgh, Pennsylvania; University of Pittsburgh, Pittsburgh, Pennsylvania; University of Pittsburgh, Pittsburgh, Pennsylvania; University of Pittsburgh, Pittsburgh, Pennsylvania; University of Pittsburgh, Pittsburgh, Pennsylvania; University of Pittsburgh School of Medicine, Pittsburgh, Pennsylvania

## Abstract

**Background:**

*E. faecium* is a common hospital-acquired bacteria in the United States, with a high prevalence of vancomycin resistance seen within this species. Hospital-adapted vancomycin-resistant *E. faecium* (VREfm) lineages, such as ST17 and ST117, contribute to the spread and persistence of VREfm within healthcare systems. Although the dynamics of these lineages within healthcare systems have been characterized, little is understood as to what drives strain replacement in VREfm.

**Methods:**

Comparative genomic analyses were performed on 718 clinical VREfm isolates collected between January 2017 and December 2022 at a large tertiary care center as part of the Enhanced Detection System for Hospital-Associated Transmission (EDS-HAT). Clusters of potential healthcare-associated transmission were determined using split kmer analysis (SKA) with a threshold of 20 single nucleotide polymorphisms (SNPs). Spot killing assays were performed using representative ST17 and ST117 isolates.

**Results:**

Of 718 clinical isolates, most were collected from urine (42%) followed by blood (23%), wounds (19%), and tissue (10%). Nearly all isolates (92%) belonged to 11 well characterized, hospital-adapted VREfm lineages (Figure 1). In total, 116 potential transmission clusters were identified containing 62% of isolates collected. Prior to 2019, lineage ST17 was dominant, but this lineage was replaced by the emergence of ST117 beginning in 2019 (Figure 2). Comparative genomic analyses revealed minimal differences in accessory genes between these lineages, including antimicrobial resistance genes (ARGs) and virulence factors. Spot killing assays showed that a lineage ST117 isolate inhibited the growth of a lineage ST17 isolate *in vitro* (Figure 3).

**Figure 1:**
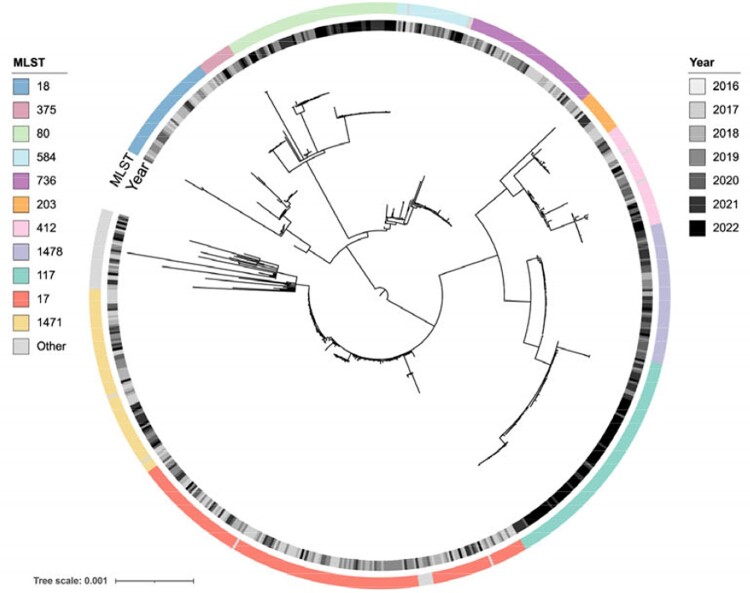
Phylogeny of 718 Vancomycin-Resistant E. faecium isolates.

The core genome phylogenetic tree was constructed using RAxML-HPC using a core genome alignment from Roary. The tree is midpoint-rooted. Isolates are colored by multi-locus sequence type (MLST) and year of culture.

**Figure 2:**
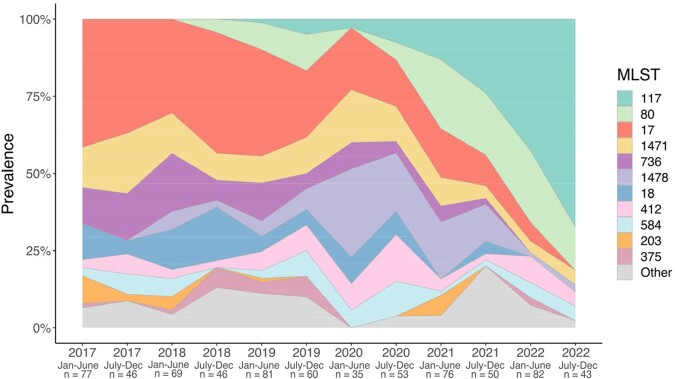
Biannual temporal lineage analysis.

Datapoints represent the distribution of multi-locus sequence types (MLST) collected within 6-month windows throughout the study period. The number of isolates (n) is indicated for each timeframe.

**Figure 3:**
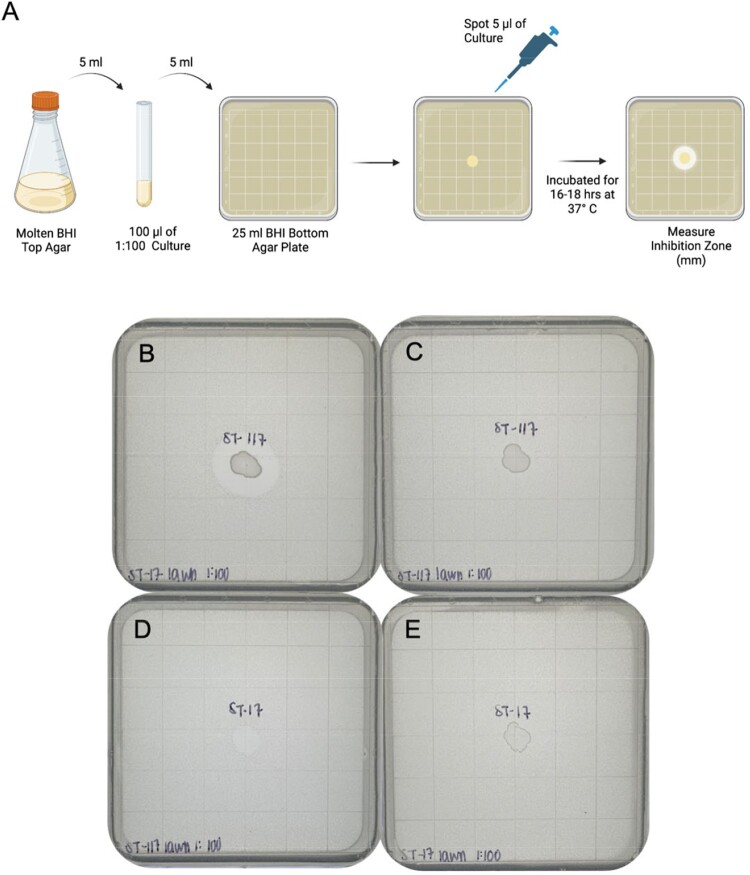
Emergent lineage ST117 inhibits growth of previously dominant, historical lineage ST17.

A) Experimental design of spot killing assay. Molten Brain Heart Infusion (BHI) top agar was mixed with 100 µL of 1:100 overnight culture of a representative ST117 or ST17 isolate and plated onto 25 mL of solidified BHI bottom agar. After the top agar set, 5 µL of the overnight cultures of ST17 and ST117 were spotted onto the bacterial lawns. Plates were incubated overnight, and inhibition zones were measured in millimeters (mm). Plates show spot killing assay of representative ST117 and ST17 isolates (B-E). B) Lawn of ST17 exposed to ST117 spot. C) Lawn of ST117 exposed to ST117 spot. D) Lawn of ST117 exposed to ST17 spot. E) Lawn of ST17 exposed to ST17 spot.

**Conclusion:**

This study provides insight into the temporal population dynamics and healthcare-associated transmission patterns of VREfm within a large tertiary care facility. We demonstrate that the 11 most prominent VREfm lineages share highly similar ARG and virulence factor profiles, which may enable persistence in the nosocomial niche. Additionally, we find evidence that emergence of specific lineages, such as ST117, may be driven by direct growth inhibition of lineages that were previously dominant in our setting.

**Disclosures:**

**Graham M. Snyder, MD, SM**, Infectious Diseases Connect: Advisor/Consultant **Alexander Sundermann, DrPH, CIC, FAPIC**, OpGen: Honoraria

